# Multiple Bronchogenic and Gastroenteric Cysts Arising from the Stomach in a Patient with Abdominal Pain

**DOI:** 10.1155/2015/601491

**Published:** 2015-07-05

**Authors:** Maykong Leepalao, Jessica Wernberg

**Affiliations:** ^1^Department of General Surgery, Marshfield Clinic, Marshfield, WI 54449, USA; ^2^Department of Surgical Oncology, Marshfield Clinic, Marshfield, WI 54449, USA

## Abstract

Bronchogenic cysts arising from the stomach are uncommon. We discuss a young female patient with presumed enteric duplication cysts who was found to have three bronchogenic and gastroenteric cysts upon pathologic review. We discuss the pathophysiology of bronchogenic cysts and their malignant potential.

## 1. Introduction

Bronchogenic cysts arising from the stomach are a relatively rare entity. There have been reported cases of intra-abdominal bronchogenic cysts as early as fifty years ago with less than 25 published reports in the literature. This report highlights a rare case of three bronchogenic cysts arising in the gastric wall of an adult patient.

## 2. Background

A 29-year-old female who had experienced severe left upper quadrant pain during pregnancy presented to our clinic. During her pregnancy, she underwent imaging which demonstrated several large cystic structures felt to be arising from the stomach. These were presumed to be enteric duplication cysts. Due to the symptomatic nature, diagnostic insecurity, and concern over malignant potential of these cysts, resection was recommended.

She did not have any other pertinent medical history. She ran cross country in high school and was healthy. She had no other issues with her prior pregnancies. She was a nonsmoker.

On physical exam, she had palpable fullness in her left upper quadrant but was otherwise nondistended, soft, and nontender. Pertinent preoperative laboratory evaluation revealed no abnormal findings.

**Figure 1:**
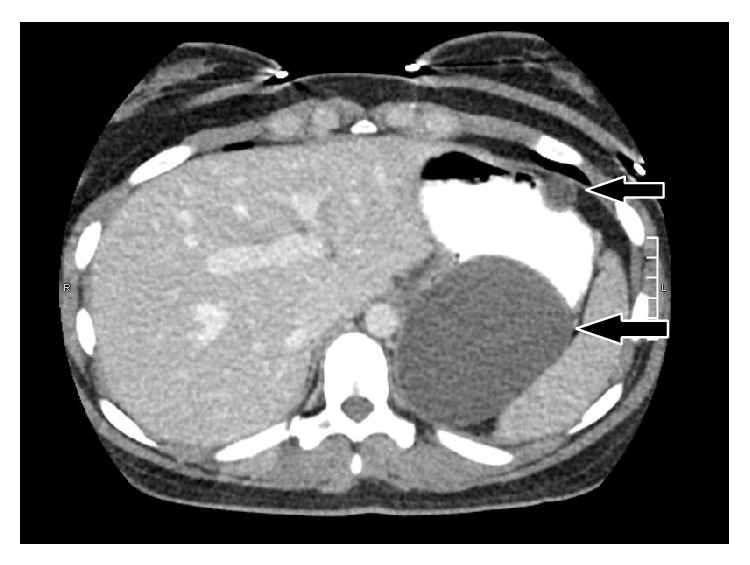
Computed tomography of intra-abdominal cysts. Arrows point to multiple cysts arising from the stomach wall (axial).

**Figure 2:**
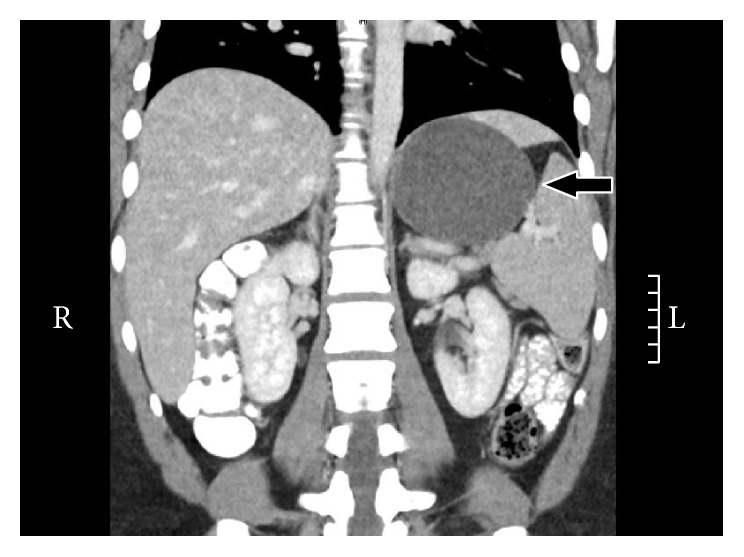
Computed tomography of intra-abdominal cysts. Arrow points to bronchogenic cyst arising from the stomach wall (coronal).

CT imaging showed three benign appearing well-demarcated thin-walled simple cystic masses in the left upper abdomen all having a mass effect on the stomach. The cysts measured 9.2 × 6.6 cm, 1.8 × 1.7 cm, and 3.0 × 2.8 cm and were located along the posterior aspect of the upper stomach, anterolateral upper abdomen, and greater curvature of the stomach, respectively (Figures 1 and 2). There had been a slight interval growth from a CT one year earlier.

Patient proceeded to the operating room where she underwent wedge resection via an upper midline incision of all three cysts with no complications. Intraoperatively, the cysts did not communicate with the gastric lumen but arose from the gastric wall. They were all soft and filled with crystalline, particulate-laden fluid.

**Figure 3:**
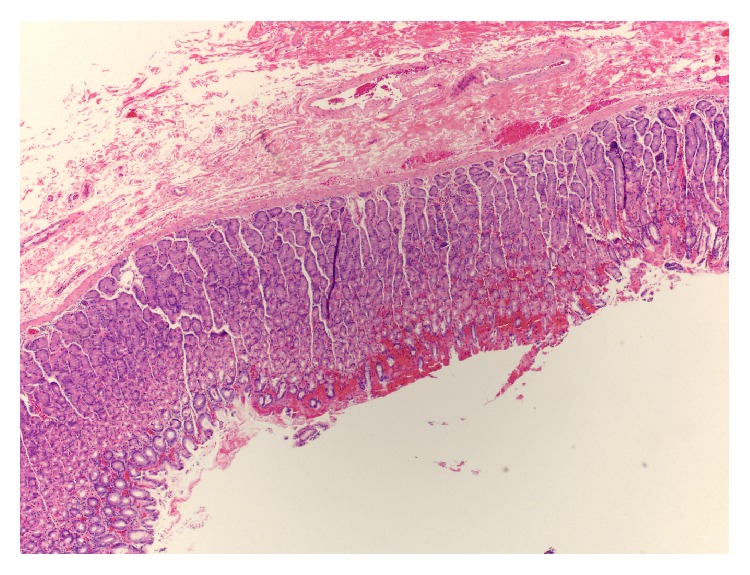
Histological H&E stain. Cystic lesion from greater curvature of stomach showing benign developmental cyst with smooth muscle wall and lined by respiratory ciliated epithelium. 40x magnification.

**Figure 4 fig4:**
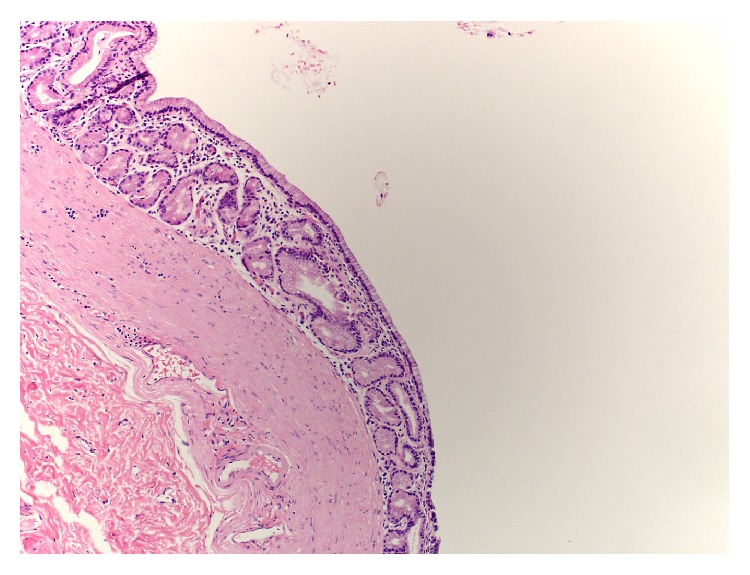
Histological H&E stain. Cystic lesion near the greater curvature of the stomach demonstrating benign developmental cyst lined by mucinous glandular epithelium resembling the epithelium of the stomach and respiratory epithelium. 100x magnification.

**Figure 5:**
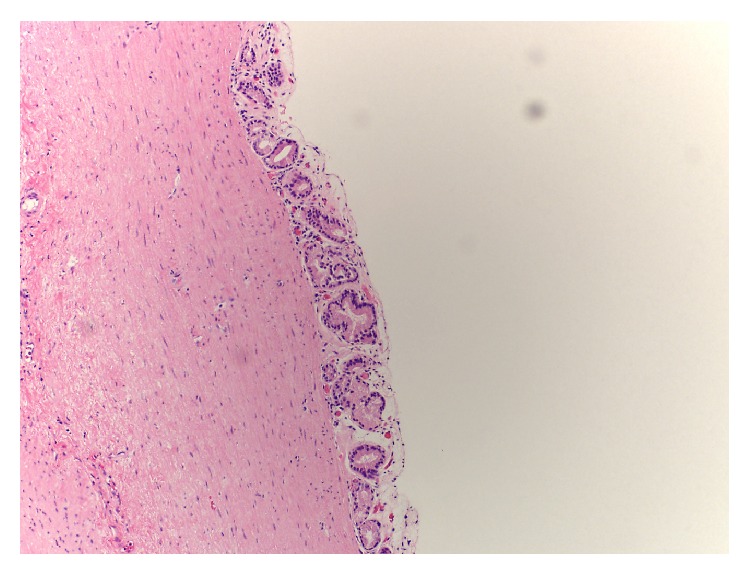
Histological H&E stain. Cystic lesion from the gastric cardia showing benign developmental cyst lined by mucinous columnar and respiratory epithelium with smooth muscle in the cyst wall. 100x magnification.

Pathology demonstrated benign developmental, thin-walled cysts with a smooth muscle wall. These were lined by respiratory ciliated and mucinous glandular epithelium resembling the epithelium of the stomach and respiratory system consistent with bronchogenic and combined bronchogenic/gastroenteric cysts (Figures 3 [Fig fig4]–5).

Given the pathology, a CT chest was completed and showed left lower lobe partial bronchial agenesis. There were no pulmonary cystic lesions. The patient, however, remained asymptomatic with no signs of respiratory difficulty or hypoxia and no further workup was done.

## 3. Discussion

There is a paucity of reported cases of intra-abdominal bronchogenic cysts. Our case outlines a unique case of three symptomatic bronchogenic or mixed bronchogenic/enteric gastric cysts. Gensler et al. reported the first case of an intramural gastric cyst in 1966 that was composed of ciliated pseudostratified columnar epithelium with focal squamous metaplasia. Since then, review of the literature reveals less than thirty case reports of single bronchogenic cysts located in the gastric mucosa [1–3].

Bronchogenic cysts typically arise from the foregut during embryological development in the 3rd to 7th week of life [4–10]. Esophageal epithelium undergoes a transient stage of cilia formation during the tenth week of gestation [5, 11] before differentiating into the usual squamous epithelium. This could potentially explain the pathophysiological mechanism for the presence of respiratory epithelium in the proximal gastrointestinal tract [5]. Congenital bronchogenic anomalies are more commonly found in the mediastinum, typically esophagus, or retroperitoneal space [12–16]. Bronchogenic cysts have also been reported to have been found on the skin [17] and diaphragm [18–23] and within the pericardium [24].

Patients have been reported to present with symptoms ranging from reflux to abdominal pain with some having no symptoms at all [6, 25]. Treatment has ranged from observation to aspiration to resection [4, 21, 26]. Patients have reported recurrence of cysts after aspiration [17]. Regardless, the majority of patients appeared to have undergone resection. The reported patient experienced abdominal pain during pregnancy with resolution after delivery, possibly due to mass effect. A hormonal component could not be excluded.

There have been a few published case reports of bronchogenic cysts involved with adenocarcinoma [26], bronchioloalveolar carcinoma, neuroblastoma [27], and rhabdomyosarcoma; however, there is minimal data in the literature to suggest oncologic potential for bronchogenic cysts [28, 29]. Most bronchogenic cysts are found incidentally and resected at the time. Vazquez et al. describe a case of a bronchogenic cyst that was found at the same time as a neuroblastoma in a pediatric patient. These were resected at separate procedures. They discuss the genetic basis for this association with speculations on oncogene mutations [27]. Sullivan et al. reported a case of adenocarcinoma arising from a retroperitoneal bronchogenic cyst. In that case, ciliated columnar epithelium was not present. Furthermore, some studies have suggested that loss of epithelial lining is associated with malignancy [26]. Our case study did have ciliated respiratory epithelium with no evidence of malignancy. However, the association between malignancy and bronchogenic cysts remains unclear.

This case highlights a rare finding of multiple bronchogenic cysts arising from the gastric wall. Clearly, more investigation needs to be done to further understand the pathophysiology of these congenital bronchogenic cysts. Symptomatic or incidentally discovered cystic lesions in the foregut are generally felt to be benign. Symptomatic lesions probably warrant resection, especially if there is any diagnostic insecurity. There are occasional reports of bleeding, ulceration, or obstruction [5, 24, 30–34], and, depending on the clinical situation, resection rather than continued observation may be appropriate.
